# Mouse model hints at pharmacogenomic strategies for stroke treatment in patients possessing a risk variant

**DOI:** 10.1172/JCI173932

**Published:** 2023-09-15

**Authors:** Douglas E. Vaughan

It has been over three decades since the thrombin receptor was cloned and its mechanism of action revealed (1). We now know that thrombin-activated G-protein coupled receptors (PARs) are a group of proteins that play a role in various physiological processes, including stroke. When thrombin, a key enzyme involved in blood clotting, binds to PARs, it triggers a cascade of cellular events. PAR-1 and PAR-4 are the two main thrombin-activated receptors relevant to stroke.In stroke, the activation of PARs by thrombin can contribute to protective and detrimental effects (2). On one hand, PAR activation can promote neuroprotection, tissue repair, and angiogenesis that may aid in stroke recovery. On the other hand, excessive PAR activation can lead to inflammation, blood-brain barrier disruption, and neuronal damage, worsening stroke outcomes. Notably, PAR-1 activation can exacerbate brain injury in stroke by promoting inflammation and blood-brain barrier dysfunction, while PAR-1 blockade has potential therapeutic benefits in reducing stroke-related damage and improving outcomes.As for PAR-4, its role in stroke is less well-defined. PAR-4 activation may contribute to platelet activation and thrombosis, which could worsen stroke outcomes. However, further investigations are needed to fully understand the impact of PAR-4 in stroke pathophysiology.

In this issue of the *JCI*, Denorme and colleagues (3) investigated the potential contribution of a known PAR-4 genetic variant with high prevalence in patients with African ancestry to ischemic stroke (IS). The F2RL3 gene that encodes PAR harbors a functional genetic variant, rs773902 A/G (encoding Thr120/Ala120, respectively) and is associated with augmented platelet aggregation (4). The A allele frequency is more common in Black individuals, and Black individuals have a higher incidence of ischemic stroke than White individuals. To examine this subject, the authors engineered an elegant model of human PAR4 (hPAR4) that expressed the normal allele and the variant in mice using a CRISPR-Cas9 gene editing approach. The major findings were notable — mice carrying the human PAR4 risk allele had worse stroke outcomes, which correlated with more platelet-neutrophil aggregates and neutrophil extracellular traps (NETs) in brain tissue than mice with the lower-risk allele. Moreover, mice carrying the high-risk allele responded well to pharmacological PAR4 inhibition and to P-selectin blockade in terms of stroke outcomes. However, the mice did not respond well to contemporary anti-platelet therapies, which instead promoted cerebral hemorrhage in the stroke model. Finally, human population data indicated that Black individuals carrying the high-risk allele were more prone to ischemic stroke and worse neurological outcomes, and human platelets carrying the high-risk allele induced greater NET formation ex vivo. These findings are important because they expand upon the current understanding of the PAR4 Ala120Thr variant in vivo and its implications for stroke outcomes. They also provide unexpected and valuable pharmacogenomic hints into therapeutic approaches that may provide better outcomes for stroke patients carrying the risk variant (Thr120) than traditional anti-platelet therapies.

## Version 1 09/15/2023

Electronic publication

## References

[1] Vu TK (1991). Molecular cloning of a functional thrombin receptor reveals a novel proteolytic mechanism of receptor activation. Cell.

[2] Ye F (2021). The role of thrombin in brain injury after hemorrhagic and ischemic stroke. Transl Stroke Res.

[3] Denorme F The predominant PAR4 variant in individuals of African ancestry worsens murine and human stroke outcomes. J Clin Invest.

[4] Edelstein LC (2014). Common variants in the human platelet PAR4 thrombin receptor alter platelet function and differ by race. Blood.

